# Evaluation of aspartate aminotransferase to platelet ratio index and fibrosis 4 scores for hepatic fibrosis assessment compared with transient elastography in chronic hepatitis C patients

**DOI:** 10.1002/jgh3.12219

**Published:** 2019-06-26

**Authors:** Pimsiri Sripongpun, Pisit Tangkijvanich, Watcharasak Chotiyaputta, Phunchai Charatcharoenwitthaya, Roongruedee Chaiteerakij, Sombat Treeprasertsuk, Chalermrat Bunchorntavakul, Abhasnee Sobhonslidsuk, Apinya Leerapun, Suparat Khemnark, Kittiyod Poovorawan, Sith Siramolpiwat, Sakkarin Chirapongsathorn, Wirichada Pan‐Ngum, Ngamphol Soonthornworasiri, Wattana Sukeepaisarnjaroen, the THASL study group

**Affiliations:** ^1^ Department of Internal Medicine Faculty of Medicine, Prince of Songkla University Hat Yai Thailand; ^2^ Center of Excellence in Hepatitis and Liver Cancer Chulalongkorn University Bangkok Thailand; ^3^ Faculty of Medicine Siriraj Hospital Bangkok Thailand; ^4^ Faculty of Medicine Chulalongkorn University Bangkok Thailand; ^5^ Department of Medicine Rajavithi Hospital Bangkok Thailand; ^6^ Faculty of Medicine Ramathibodi Hospital Bangkok Thailand; ^7^ Department of Internal Medicine Chiang Mai University Chiang Mai Thailand; ^8^ Department of Medicine Bamrasnaradura Infectious Diseases Institute Nonthaburi Thailand; ^9^ Department of Clinical Tropical Medicine Mahidol University Bangkok Thailand; ^10^ Department of Internal Medicine Thammasat University Pathumthani Thailand; ^11^ Department of Medicine Phramongkutklao Hospital Bangkok Thailand; ^12^ Department of Tropical Hygiene Mahidol University Bangkok Thailand; ^13^ Department of Medicine Khon Kaen University Khon Kaen Thailand

**Keywords:** aspartate aminotransferase to platelet ratio index, biomarker, fibrosis, fibrosis 4, hepatitis C, noninvasive, transient elastography

## Abstract

**Background and Aim:**

Fibrotic stage (FS) assessment is essential in chronic hepatitis C treatment cascade. Liver stiffness measurement (LSM) using transient elastography (TE) is reliable and correlated with liver biopsy. However, TE may not be widely available. This study aimed to evaluate the diagnostic performances of aspartate aminotransferase to platelet ratio index (APRI) and fibrosis 4 (FIB‐4) scores compared with TE.

**Methods:**

We conducted a multicenter, cross‐sectional study, including all chronic hepatitis C virus (HCV) monoinfection patients with successful and reliable LSM, at 10 centers in Thailand from 2012 to 2017. Characteristics and laboratory data within 3 months of TE were retrospectively reviewed. Using TE as a reference standard, the diagnostic performances of APRI and FIB‐4 were evaluated. TE cut‐off levels of 7.1 and 12.5 kPa represented significant fibrosis (SF) and cirrhosis, respectively.

**Results:**

The distribution of FS by TE in 2000 eligible patients was as follows: no SF 28.3%, SF 31.4%, and cirrhosis 40.3%. APRI ≥ 1 provided 70.1% sensitivity and 80.6% specificity, with an area under the receiver operator characteristics curve (AUROC) of 0.834 for cirrhosis. The specificity increased to 96.3% when using a cut‐off level of APRI ≥ 2. FIB‐4 ≥ 1.45 provided a sensitivity, specificity, and AUROC of 52.4%, 91.0%, and 0.829 for cirrhosis, respectively. For SF, APRI performed better than FIB‐4, with an AUROC of 0.84 *versus* 0.80 (*P* < 0.001). APRI score < 0.5 and FIB‐4 score > 1.45 yielded sensitivities of 82.3% and 74.4% and specificities of 65.4% and 69.8%, respectively.

**Conclusions:**

APRI and FIB‐4 scores had good diagnostic performances for FS assessment compared with TE, especially for cirrhosis. APRI may be used as the noninvasive assessment in resource‐limited settings for HCV patients’ management.

## Graphical abstract

Using transient elastography as the standard reference, aspartate aminotransferase to platelet ratio index (APRI) and fibrosis 4 (FIB‐4) performed well in the prediction of cirrhotic status in chronic hepatitis C patients, but APRI was better than FIB‐4 for the prediction of significant fibrosis (SF). The high cut‐off values of APRI proposed by World Health Organization (WHO) for both SF and cirrhosis yielded a very high positive predictive value to determine those conditions, but at the WHO low cut‐off values, the negative predictive values were not satisfactorily high enough to rule out such conditions

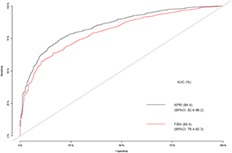

## Introduction

The assessment of hepatic fibrosis stage is essential in the management of patients with chronic liver diseases of various etiologies. For example, the determination of cirrhotic status (F4 by METAVIR staging) triggers the management bundle of cirrhosis care, such as surveillance for portal hypertension complications and hepatocellular carcinoma (HCC)^1^; significant fibrosis (SF) (≥F2 by METAVIR staging) should be present prior to the initiation of antiviral treatment in chronic hepatitis B patients in many guidelines,^2^, ^3^, ^4^ and it is also a significant surrogate marker for the progression to cirrhosis in other chronic liver diseases such as chronic hepatitis C (CHC), non‐alcoholic fatty liver disease (NAFLD),^5^ etc. In CHC patients, in compliance with the 2030 World Health Organization (WHO) global hepatitis C virus (HCV) elimination target,^6^ despite the fact that all CHC patients should be treated, treatment prioritization for patients with SF (≥F2 fibrosis by METAVIR score) is still the mainstay of resource allocation in low‐ and middle‐income countries.^7^

Although liver biopsy is the gold standard for the histological assessment of fibrosis status,^8^ it is an invasive method, with uncommon but potentially life‐threatening complications. Recently, liver stiffness measurement (LSM) using transient elastography (TE) has become the most common noninvasive method to assess hepatic fibrosis in many countries.^9^, ^10^ TE is widely used because of its reproducibility and excellent inter‐ and intraobserver agreement, and it has been validated in enormous numbers of studies, which showed a very good correlation to histological assessment by liver biopsy.^11^, ^12^, ^13^, ^14^ However, TE has some disadvantages; it requires a specific device that is costly for many centers, and failure to obtain the measurement has been reported in some particular groups of patients, for instance, ascites, obesity, narrow rib space, etc.^8^

Another currently available noninvasive method is the serum biomarkers of liver fibrosis.^8^ Several biomarkers have been reported in literature; some are patented, for example, Fibrotest® and Hepascore, while some are composed of unpatented clinical and laboratory parameters. Aspartate aminotransferase to platelet ratio index (APRI) and fibrosis 4 (FIB‐4) scores are among the unpatented biomarkers using clinical and laboratory values that are routinely monitored or so‐called “bedside” investigations in chronic hepatitis patients, which make these scoring systems more likely to be applicable in general practice. APRI and FIB‐4 scores are also recommended by WHO to be used in the assessment of hepatic fibrosis in resource‐limited settings where cost and availability of TE are the barriers.^7^ FIB‐4 and APRI scores have been validated in some specific groups of patients and showed a good correlation with liver biopsy.^15^, ^16^, ^17^, ^18^, ^19^ However, the data regarding diagnostic performances of FIB‐4 and APRI score compared with TE are limited. This study aims to evaluate diagnostic performances of FIB‐4 and APRI scores compared with fibrosis staging by TE in Thai CHC patients.

## Methods

### *Study design and data collection*

This is a multicenter, cross‐sectional study from 10 centers across different regions of Thailand. We included All CHC patients who underwent successful LSM using Fibroscan® (Echosens, France) from the transient elastography database in each center in 2012–2017, who were not treated with Peginterferon and Ribavirin or direct antiviral agents (DAA) at the time of LSM. Chronic HCV infection was defined by the presence of a serum HCV antibody (anti‐HCV+) and detectable viremia by HCV RNA. Exclusion criteria were: (i) known coinfection with the human immunodeficiency virus (HIV) or hepatitis B virus (HBV); (ii) no available laboratory results of aspartate aminotransferase (AST), alanine aminotransferase (ALT), and platelet level within 3 months of LSM; (iii) patients with coexisting diseases of either active hemolysis at the time of AST/ALT measurement, immune‐mediated thrombocytopenia, or hematological malignancies that may interfere with the results of APRI and/or FIB‐4 calculation; (iv) patients with AST or ALT > 5 times above the upper normal limit (ULN); and (v) the subsequent TE measurement(s) in the same patients after the first enrollment (to avoid repeated measurement data in the same patient that will subsequently generate correlated data among independent observations).

Data from medical records were retrieved for baseline characteristics, HCV genotype, HCV RNA, and laboratory results within 3 months of LSM. The TE results were retrieved from each center's TE database.

The study protocol was approved by the Human Research Ethics Committee (HREC) of all participating centers. Informed consent was waived as the data were retrospectively retrieved and analyzed in a deidentified format, and the case record form was also approved by the HREC of all participating centers.

### *Transient elastography and biomarkers*

All transient elastography was carried out with a Fibroscan® (Echosens, France), and successful TE was defined if the following criteria were fulfilled^3^: (i) a number of valid shots of at least 10; (ii) a success rate (the ratio of valid shots to the total number of shots) >60%; and (iii) an interquartile range (IQR) < 30% of the median liver stiffness measurements (M) value (IQR/M < 0.30).

Using TE as the reference standard, the diagnostic performances of APRI and FIB‐4 scores were then evaluated. The TE cut‐off levels of 7.1 kPa and 12.5 kPa were defined as characteristic of SF (F ≥ 2) and cirrhosis (F = 4),^20^ respectively. APRI and FIB‐4 scores were calculated from baseline laboratory data using the standard formula^15^, ^17^ shown below. The normal upper limit of AST levels was defined according to local laboratory values in each center. The previous recommended cut‐off values for SF (APRI 0.5, 1.5, FIB‐4 1.45) and cirrhosis (APRI 1, 2, FIB‐4 1.45, 3.25) were used to define the positive results of tests.^7^, ^15^, ^17^

$$\text{APRI}=\frac{\mathrm{AST}\left[U/\mathrm{L] / AST upper limit of normal [U/L}\right]}{\text{platelet}\left[10^{9}/L\right]}\times 100$$

$$\mathrm{FIB}‐4\;\text{score}=\frac{\mathrm{age}\;\left[\mathrm{age}\right]\times \mathrm{AST}\;\left[U/L\right]}{\text{platelet}\;\left[10^{9}/L\right]\times \sqrt{\mathrm{ALT}\left[U/L\right]}}$$

### *Statistical analysis*

Descriptive statistics was used for baseline demographic data. Quantitative measurements were shown as mean ± SD or median with IQR according to the distribution of observed values. Categorical variables were expressed using numbers and percentages. Correlation analysis was used to compare the liver stiffness (in kPa) with APRI and FIB‐4 scores. Using TE as the reference standard, the diagnostic performances of APRI and FIB‐4 scores were evaluated through the calculation of the area under the receiver operator characteristics curve (AUROC) and were also reported as sensitivity and specificity of different cut‐off values. DeLong's test was used to compare between two receiver operator characteristics curve (ROC) curves of APRI and FIB‐4 for cirrhosis and SF stage.

## Results

Of 2242 patients from 10 centers’ TE database, 242 patients were excluded according to exclusion criteria (known coinfection with HIV or HBV = 70, no available AST or ALT or platelet results = 115, coexisting significant hematologic disease = 1, and AST/ALT level > 5 times ULN = 56). Hence, a total of 2000 eligible patients were included in the analysis. The demographic and clinical characteristics of the study population are shown in Table 1. The mean age was approximately 52 years, with a slight male predominance (59%); the majority of patients had HCV RNA of less than 600,000 IU/mL, and around 40% of the entire study population had cirrhosis according to TE criteria.

**Table 1:** Baseline characteristics of enrolled patients (*n* = 2000)

Characteristics	*n* (%)
Region in Thailand	
*Northern*	
Chiang Mai University	93 (4.7)
*Central*	
Siriraj Hospital	624 (31.2)
Chulalongkorn University	376 (18.8)
Rajvithi Hospital	323 (16.2)
Ramathibodi Hospital	168 (8.4)
Bamrasnaradura Infectious Diseases Institute	77 (3.8)
Thammasart University	35 (1.8)
Tropical Medicine	3 (0.2)
*Northeastern*	
Srinagarind Hospital	110 (5.5)
*Southern*	
Prince of Songkla University	191 (9.6)
Age, years (mean ± SD)	51.7 ± 10.5
Gender, male	1183 (59.2)
HCV genotype (of 1684 available data)	
Genotype 1	700 (41.6)
Genotype 3	776 (46.1)
Genotype 6	193 (11.5)
Genotype 2 or 4	15 (0.8)
HCV RNA (of 1610 available data)	
≤600 000 IU/mL	1363 (84.7)
≥600 000 IU/mL	247 (15.3)
Liver stiffness, kPa (median, IQR)	10.2 (6.7, 17.3)
AST level, U/L (median, IQR)	52 (33, 81)
ALT level, U/L (median, IQR)	62 (36, 102)
Platelet, *10^9^/L (mean ± SD)	190 ± 75.55
Fibrosis stage by TE	
No significant fibrosis (F0‐1)	566 (28.3)
Significant fibrosis (F2‐3)	628 (31.4)
Cirrhosis (F4)	806 (40.3)

ALT, alanine aminotransferase; AST, aspartate aminotransferase; HCV, hepatitis C virus; IQR, interquartile range; TE, transient elastography.

The APRI and FIB‐4 scores showed a significant correlation with TE results (*r* = 0.489, *P* < 0.001; and *r* = 0.519, *P* < 0.001, respectively).

### *Diagnostic performances for APRI and FIB‐4 scores in cirrhosis*

**Figure 1:**
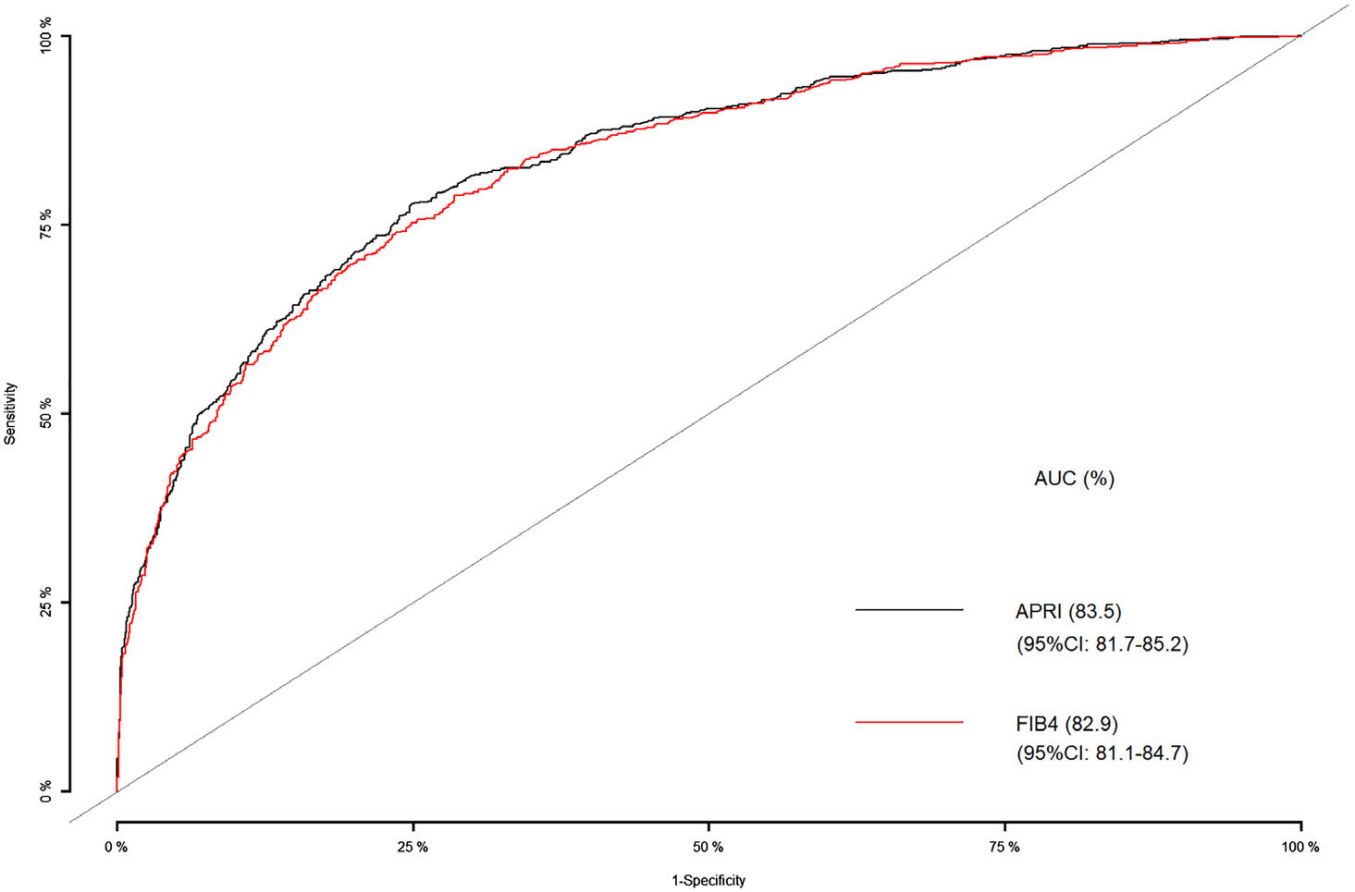
Area under the receiver operator characteristics curve analysis for the diagnosis of cirrhosis using APRI and FIB‐4 scores. AUC, area under the receiver operator characteristics curve; APRI, aspartate aminotransferase to platelet ratio index; CI, confidence interval; FIB‐4, fibrosis 4.

An ROC was created to depict the trade‐off between sensitivities and specificities of cirrhosis at different thresholds of APRI and FIB‐4 score cut‐off values. The AUROC was 0.835 for APRI (95% confidence interval [CI], 0.82–0.85) and 0.829 for FIB‐4 (95% CI, 0.81–0.85) as shown in Figure 1. There was no significant difference between AUROC of APRI and FIB‐4 for cirrhosis (*P* = 0.3721).

The sensitivity, specificity, positive predictive value (PPV), and negative predictive value (NPV) at the cut‐off points of APRI and FIB‐4 score for predicting cirrhosis are shown in Table 2. An APRI score ≥ 2 (WHO high cut‐off value for predicting cirrhosis) provided a specificity for classifying cirrhosis as high as 96.3%. However, an APRI score of less than 1 (WHO low cut‐off value for cirrhosis) provided only 70.1% sensitivity (NPV 80%) for ruling out cirrhosis. For the better sensitivity to rule out cirrhosis, we evaluated the ROC analysis and detected that a cut‐off value of APRI score < 0.5 provided better sensitivity and NPV (Table 2).

**Table 2:** Diagnostic performances of APRI and FIB‐4 scores for predicting cirrhosis

Cut‐off value	Sensitivity, % (95% CI)	Specificity, % (95% CI)	PPV, % (95% CI)	NPV, % (95% CI)
APRI ≥ 1 (WHO low cut‐off value)	70.1 (66.8–73.2)	80.6 (78.2–82.8)	70.9 (67.6–74.0)	80 (77.6–82.2)
APRI ≥ 2 (WHO high cut‐off value)	36.2 (32.9–39.7)	96.3 (95.1–97.3)	86.9 (82.8–90.3)	69.1 (66.8–71.3)
FIB‐4 ≥ 3.25	49 (45.5–52.5)	92 (90.3–93.4)	80.4 (76.7–83.9)	72.8 (70.4–75.0)
APRI ≥ 0.5 (the present study's lower cut‐off value)	90.9 (88.7–92.8)	46.1 (43.3–49)	53.3 (50.6–55.9)	88.3 (85.5–90.7)

APRI, aspartate aminotransferase to platelet ratio index; CI, confidence interval; FIB‐4, fibrosis 4; NPV, negative predictive value; PPV, positive predictive value; WHO, World Health Organization.

### *Diagnostic performances for APRI and FIB‐4 score in SF*

**Figure 2:**
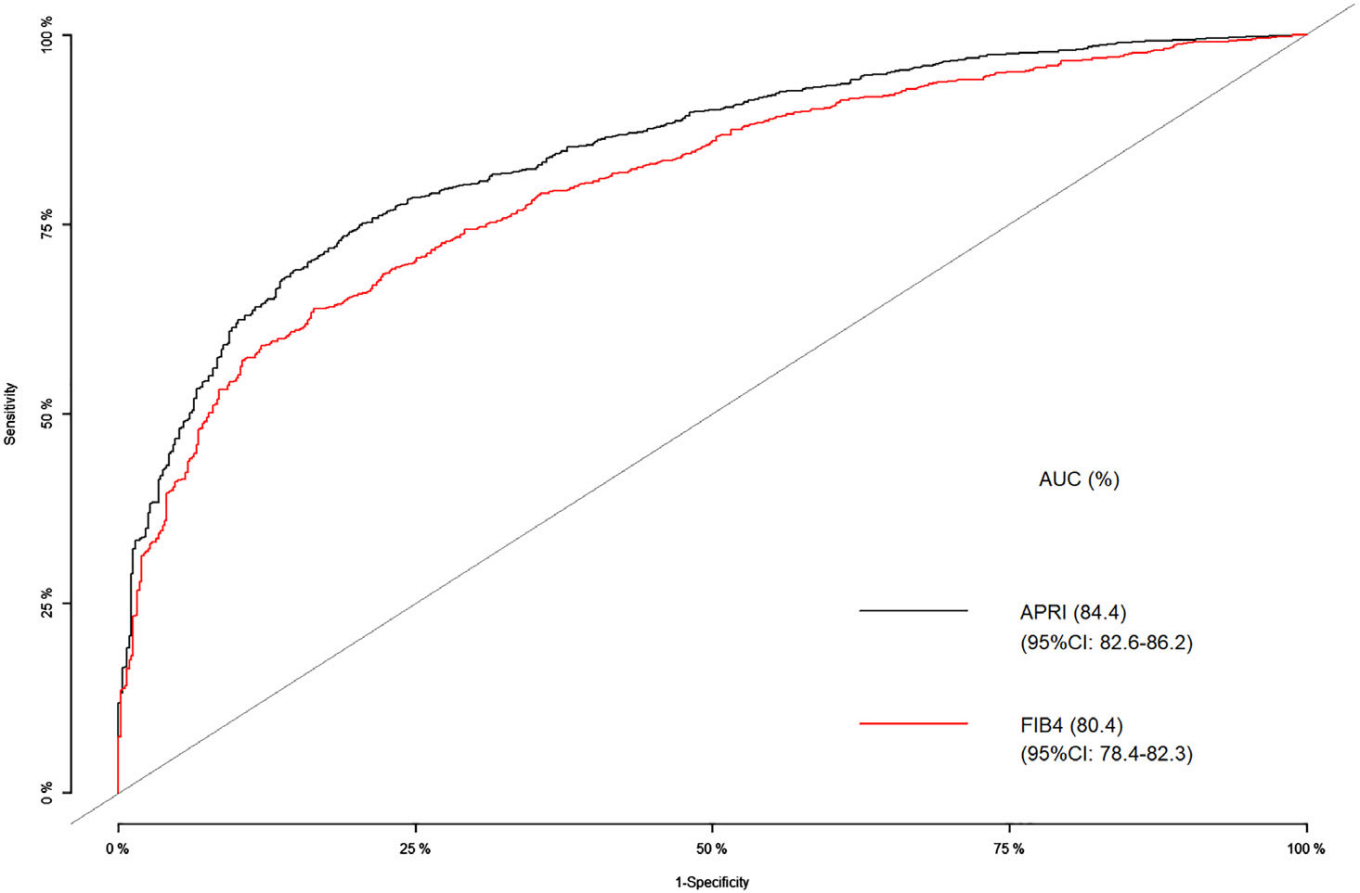
Area under the receiver operator characteristics curve analysis for the diagnosis of significant fibrosis using APRI and FIB‐4 scores. AUC, area under the receiver operator characteristics curve; APRI, aspartate aminotransferase to platelet ratio index; CI, confidence interval; FIB‐4, fibrosis 4.

For SF (TE ≥ 7.1 kPa), APRI performances were significantly better than FIB‐4 scores as the AUROCs were 0.844 for APRI (95% CI, 0.83–0.86) and 0.804 for FIB‐4 (95% CI, 0.78–0.82), *P* < 0.001 (Fig. 2). The sensitivity, specificity, PPV, and NPV at the cut‐off points of APRI and FIB‐4 scores for predicting cirrhosis are shown in Table 3. An APRI score ≥ 1.5 (WHO high cut‐off value) provided a specificity for predicting SF stage as high as 97.7%. However, an APRI score of less than 0.5 (WHO low cut‐off value) provided only 59.3% NPV for ruling out SF. For the better sensitivity to rule out SF stages in CHC patients, we evaluated the ROC analysis and detected that a cut‐off APRI score < 0.3 provided better sensitivity and NPV (Table 3).

**Table 3:** Diagnostic performances of APRI and FIB‐4 scores for predicting significant fibrosis

Cut‐off value	Sensitivity, % (95% CI)	Specificity, % (95% CI)	PPV, % (95% CI)	NPV, % (95% CI)
APRI ≥ 0.5 (WHO low cut‐off value)	82.3 (80.2–84.2)	65.5 (61.3–69.3)	85.8 (83.8–87.6)	59.3 (55.3–63.2)
APRI ≥ 1.5 (WHO high cut‐off value)	34.4 (32–37)	97.7 (96.1–98.8)	97.4 (95.7–98.6)	37 (34.6–39.5)
FIB‐4 ≥ 1.45 (WHO low cut‐off value)	74.4 (72.1–76.6)	69.8 (65.8–73.5)	86.2 (84.1–88.1)	51.8 (48.2–55.4)
FIB‐4 ≥ 3.25 (WHO high cut‐off value)	33.1 (30.7–35.6)	97.2 (95.4–98.4)	96.7 (94.8–98.1)	36.4 (34–38.9)
APRI ≥ 0.3 (the present study's lower cut‐off value)	95 (93.8–96.1)	35 (31.1–39.1)	78.7 (767–80.6)	73.6 (67.9–78.8)

APRI, aspartate aminotransferase to platelet ratio index; CI, confidence interval; FIB‐4, fibrosis 4; NPV, negative predictive value; PPV, positive predictive value; WHO, World Health Organization.

## Discussion

Hepatic fibrosis assessment is mandatory in the clinical management of patients with CHC in terms of both cirrhosis care and prioritizing patients with SF who are at high risk of progression to cirrhosis to be treated with DAAs, especially in low‐ and middle‐income countries where treatment of all CHC patients, despite being an ultimate goal, might not be practical in the real‐world practice. Currently, TE plays a major role in hepatic fibrosis assessment in many countries due to its excellent correlation with liver biopsy for each stage of fibrosis and poses an extremely low risk of procedure‐related complication. TE is now also an acceptable alternative standard for hepatic fibrosis assessment in CHC patients by many guidelines.^21^, ^22^, ^23^ Notwithstanding the benefits of TE, in a resource‐limited setting, the cost and the access to the centers where equipment is available are still the barriers to TE. WHO recommends using APRI and FIB‐4 scores to assess hepatic fibrosis in resource‐limited setting rather than TE; however, with a low‐quality of evidence.^7^ In this study, we conducted a large nationwide cross‐sectional study to evaluate the bedside noninvasive biomarkers APRI and FIB‐4 scores compared with TE in the assessment of cirrhosis and SF stage in CHC patients.

We found that both APRI and FIB‐4 performed well in predicting cirrhosis with an AUROC of 0.835 and 0.829, respectively, and the results are similar to the AUROC of APRI and FIB‐4 score for cirrhosis in the previous meta‐analysis using liver biopsy as the reference standard.^24^ However, regarding the prediction of SF status, the APRI score yielded a significantly better diagnostic performance over FIB‐4 score with the AUROC of 0.844 and 0.804 (*P* < 0.001), respectively. Both AUROCs are numerically higher than the previous meta‐analysis;^24^ nevertheless, both low and high cut‐off values of APRI score provide sensitivity and specificity similar to the previous report. Given the simpler calculation formula and better diagnostic performance, we suggest that the APRI score be used for hepatic fibrosis assessment in the management of CHC patients over the FIB‐4 score.

For clinical application, the prespecified high cut‐off points proposed by WHO yielded satisfactory results for defining both cirrhosis and SF. In order to start treatment with DAA in CHC patients, an APRI ≥ 1.5 (high cut‐off value for ≥F2 by WHO) yielded a PPV of 97.4% to ensure that the patients had SF, and an APRI score ≥ 2 (high cut‐off value for cirrhosis by WHO) provided a specificity of 96.3%, which could aid the primary doctors to trigger cirrhosis care bundles for the patients right away including HCC surveillance. Nonetheless, the prespecified WHO criteria (low cut‐off) did not yield satisfactory high sensitivity and NPV as the NPV were only 80 and 59.3% to rule out cirrhosis and SF, respectively, meaning that a significant proportion of patients may not receive the appropriate management if these low cut‐off values are used as a screening tool to rule out cirrhosis or SF status.

For a better sensitivity to rule out cirrhosis and SF, in our study, we have found that lower cut‐off values of APRI of 0.5 and 0.3 yielded a sensitivity of 90.0 and 95% to rule out cirrhosis and SF, respectively. The result of the lower cut‐off value of APRI of 0.5 to exclude cirrhosis in our study is similar to the recent study from Australia,^25^ where a cut‐off level of 0.49 yielded an impressive NPV of 99%. However, because we used TE as the reference standard in our research, and it was not a gold standard, this lower cut‐off value of 0.5 and 0.3 to rule out cirrhosis and SF cannot be recommended at this time. Further studies to define the appropriate low cut‐off values as a screening tool to rule out cirrhosis and SF and the validation of those cut‐off values are needed.

The strengths of our study are as follows: to date, this is the study including the largest number of CHC patients to evaluate the diagnostic performances of APRI and FIB‐4 scores for fibrosis assessment, and we included the data from all major centers across the country with a wide spectrum of liver diseases in CHC patients in Thailand. Nonetheless, we acknowledged that there are some limitations to our analysis. First, the data were collected from participant centers, which are mostly tertiary care centers; hence, there might be referral bias of the patients leading to a greater proportion of cirrhosis observed in our study. Second, we use TE as the reference standard, not the gold standard, of liver biopsy; thus, some variabilities may exist. However, TE has been validated in enormous numbers of studies, which showed a very good correlation to histological assessment by liver biopsy.^11^, ^12^, ^13^, ^14^ Furthermore, we also obtained only successful TE data using predefined criteria and exclude patients with AST or ALT > 5 times above ULN, which might interfere with the interpretation of fibrosis level from TE, minimizing the chance of unreliable results. Third, due to retrospective data collection, there were some missing data, for example, data regarding history of drug and herbal/alternative medicine use that might affect ALT level were not systematically obtained, and controlled attenuation parameter (CAP) for fat quantification data from Fibroscan® were not available in all patients as a result of different versions of the devices used across centers in the study. We could only exclude patients with known HIV/HBV coinfection, and the anti‐HIV and HBsAg tests might not be performed in all patients.

In conclusion, from this large cross‐sectional study, both APRI and FIB‐4 scores were found to have good diagnostic performances in the diagnosis of cirrhosis and SF stage, with a slightly but significantly better AUROC of APRI over FIB‐4 scores in the prediction of SF. We suggest that the WHO high cut‐off values could be used in determining both cirrhosis and SF stage in real‐world resource‐limited setting; however, to rule out significant liver disease, the low cut‐off values might need to be revised.
